# Assessment of Corneal Angiography Filling Patterns in Corneal Neovascularization

**DOI:** 10.3390/jcm12020633

**Published:** 2023-01-12

**Authors:** Luca Pagano, Haider Shah, Kunal Gadhvi, Mohammad Ahmad, Nardine Menassa, Giulia Coco, Stephen Kaye, Vito Romano

**Affiliations:** 1 St. Paul’s Eye Unit, Royal Liverpool University Hospital, Liverpool L7 8XP, UK; 2 Department of Biomedical Sciences, Humanitas University, Pieve Emanuele, 20090 Milan, Italy; 3 Department of Eye and Vision Science, Institute of Life Course and Medical Sciences, University of Liverpool, Liverpool L7 8TX, UK; 4 Department of Clinical Science and Translational Medicine, University of Rome Tor Vergata, 00133 Rome, Italy; 5 Eye Unit, Department of Medical and Surgical Specialties, Radiological Sciences, and Public Health, University of Brescia, Viale Europa 15, 25123 Brescia, Italy; 6 Eye Unit, ASST Spedali Civili di Brescia, Piazzale Spedali Civili, 1, 25123 Brescia, Italy

**Keywords:** corneal neovascularization, corneal vessels, CoNV, vessels filling pattern, ISNT rule

## Abstract

The purpose of the paper is to describe vascular filling patterns in corneal neovascularization (CoNV) and evaluate the effect of corneal lesion location, CoNV surface area and multi-quadrant CoNV involvement on the filling pattern. It is a retrospective study of patients who were investigated for CoNV using fluorescein angiography (FA) or indocyanine green angiography (ICGA) between January 2010 and July 2020. Angiography images were graded and analyzed multiple independent corneal specialists. The corneal surface was divided into four quadrants and patient information was obtained through electronic records. A total of 133 eyes were analyzed. Corneal lesions were located on the peripheral (72%) or central (28%) cornea. Central lesions were associated with multi-quadrant CoNV more frequently than peripheral lesions (*p* = 0.15). CoNV located within the same quadrant of the corneal lesion was often first to fill (88.4%). In multi-quadrant CoNV, the physiological inferior–superior–nasal–temporal order of filling was usually respected (61.7%). Central lesions resulted in larger CoNV surface area than peripheral lesions (*p* = 0.09). In multi-quadrant CoNV, the largest area of neovascularization was also the first to fill in (peripheral lesion 74%, central lesion 65%). Fillings patterns in healthy corneas have previously been reported. Despite CoNV development, these patterns are usually respected. Several factors that may influence filling patterns have been identified, including corneal lesion location, CoNV surface area and aetiology of CoNV. Understanding filling patterns of neovascularization allows for the identification of areas at higher risk of developing CoNV, aiding in earlier detection and intervention of CoNV.

## 1. Introduction

A unique property of the healthy cornea is its angiogenic privilege. This characteristic is sustained by local pro- and anti-angiogenic factors [1]. Insults, such as inflammation, infection or surgery, may disrupt this balance, resulting in corneal neovascularization (CoNV). New vessels typically arise from the marginal corneal vascular arcades (MC); however, they can arise from conjunctival, episcleral and iris vessels [2]. This pathological process leads to a loss of corneal transparency, localised lipid sequestration and chronic inflammation.

CoNV plays a key role in corneal transplantation and the presence of CoNV is a leading cause of corneal graft failure [3]. New vessels and accompanying lymphatics deliver allo-antigens to host lymphoid tissue, resulting in a loss of immune privilege, thus facilitating allograft rejection [4]. Prompt identification and treatment of CoNV is important in reducing the risk of corneal graft rejection and improving outcomes. Current therapies include topical or localized delivery of anti-angiogenic factors and/or mechanical disruption of vessels with modalities such as fine needle diathermy [5,6].

Although CoNV may be appreciated with slit lamp biomicroscopy, accurately defining the anatomical origin and distribution of the new vessels, as well as monitoring progression, is challenging, and not all vessels are clinically detected. Currently, anterior segment angiography is the only dynamic examination that can facilitate the analysis of normal and abnormal corneal vasculature [7,8]. Both fluorescein (FA) and indocyanine green angiography (ICGA) have been utilized—the latter providing excellent angiographic detail, even in the presence of scarring, as it is not subject to leakage [2]. Effective utilization of angiography can allow for early identification and better delineation of neovascularization, thus potentiating accurate risk assessment prior to corneal grafting, as well as aiding in treatments such as fine needle diathermy [9].

Despite being an important component of assessing CoNV, there is limited information on angiographic filling patterns. Zheng et al. described an ordered filling pattern of inferior–superior–nasal–temporal (ISNT pattern) in healthy marginal corneal arcades (MCA) [2]. There is no information, however, on the filling patterns of diseased corneas with CoNV. The purpose of this study, therefore, was to define the angiographic filling patterns of neovascularized corneas. Better understanding of filling patterns may help identify areas of the cornea at higher risk of CoNV and guide treatment.

## 2. Materials and Methods

### 2.1. Study Population

The images of patients who presented with corneal lesions with accompanying CoNV who had undergone FA and ICGA at The Royal Liverpool University Hospital between January 2010 and July 2020 were included. Data collection was initially performed from the Heidelberg OCT software and subsequently from electronic patient records. All scans categorized as ‘anterior segment angiography” on the Heidelberg OCT software were included. Angiographies demonstrating 1 or more quadrants of CoNV were studied. Anterior segment angiography performed for conjunctival lesions with localized extension onto the cornea, such as ocular surface malignancy and pterygium, were excluded. Low-quality images were excluded as detailed below. Clinical details and patients’ demographics were obtained from patients’ electronic records. No identifiable information was recorded. Ethical approval was obtained by the Stanmore Ethics Committee (15/LO/2166) and the study was conducted in accordance with the tenets of the Declaration of Helsinki.

### 2.2. Analysis of Images

Image quality was first assessed by 2 independent ophthalmologists and qualitatively graded from 0 to 4 (0 = no discernible vessels, 1 = poor vessel delineation, 2 = good vessel delineation, 3 = very good vessel delineation, 4 = excellent vessel delineation). Only images graded as 2 and above by both observers were included [7]. Patients were also excluded if discrimination of the order of CoNV filling was not possible due to blinking or loss of focus in the images/videos.

**Figure 1:**
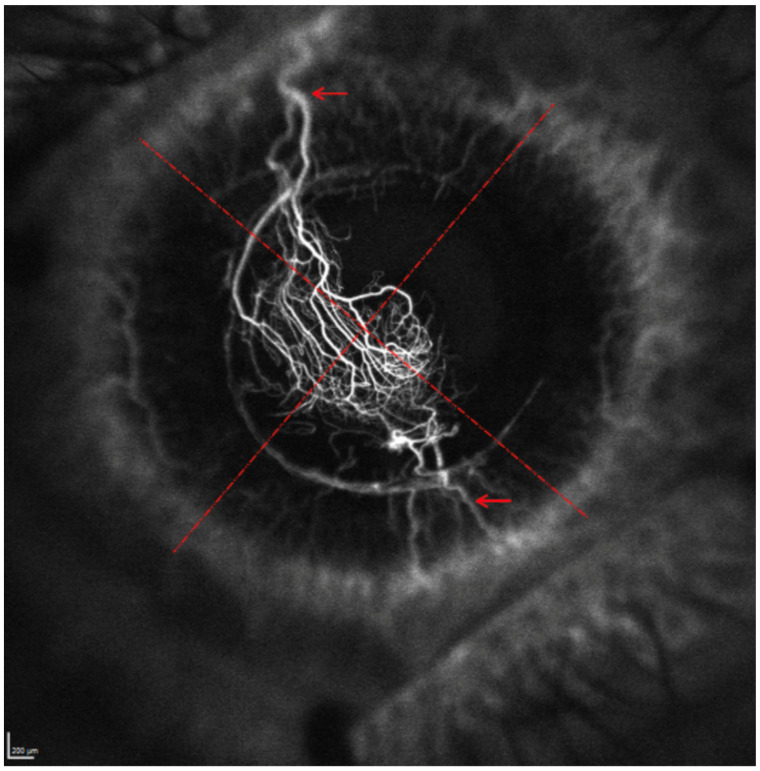
Division of cornea into four quadrants with a super-imposed cross image (dashed). The origin of the ConV is highlighted by arrows.

**Figure 2:**
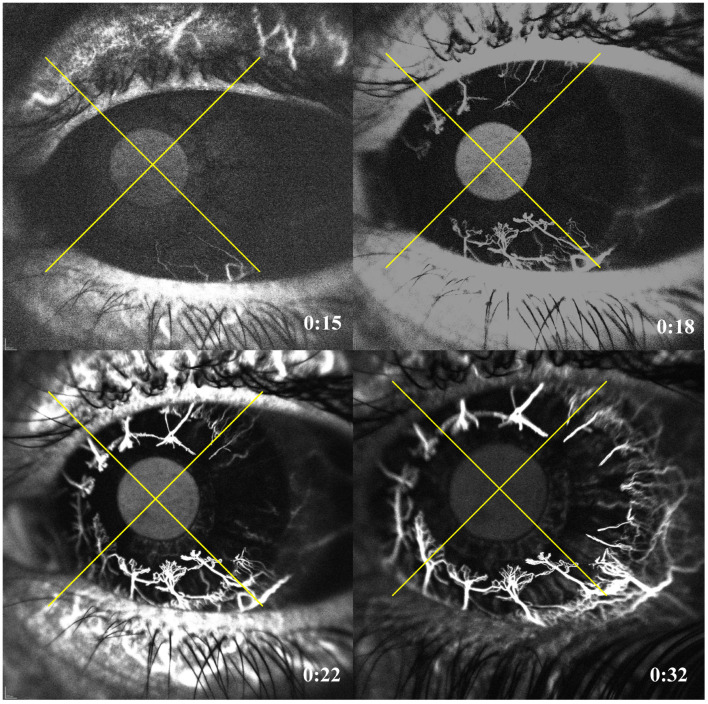
Indocyanine angiography showing a corneal neovascularization following the ISNT rule. The patient had a left eye penetrating keratoplasty that was followed by intensive 360° neovascularization. (**Top left**) shows that the first quadrant to fill is the inferior at 15 s, followed by (**top right**) the superior quadrant at 18 s, then the nasal quadrant at 22 s (**bottom left**) and ultimately the temporal quadrant at 32 s (**bottom right**).

Each cornea was divided into 4 quadrants (superior, inferior, nasal and temporal) by super-imposing a cross image (Figure 1). The location of the corneal lesion was recorded as either central or peripheral if the lesion was close to or adjacent to the limbus. Peripheral lesions were grouped according to which quadrant they were located in. FA and ICGA images and videos were reviewed by 2 independent ophthalmologists (cornea specialists) to assess: (1) the location and order of CoNV filling patterns (Figure 2) and (2) the extent of CoNV. The origin of CoNV was determined by its point of origin from the MCA. All images were assessed in the same environmental condition with dim room light and maximum computer screen lighting.

### 2.3. Statistical Analysis

Data are presented as the mean ± standard deviation (SD), or as a percentage for categorical variables.

The Chi-squared test was used to determine statistical differences between groups. The statistical analyses were performed using STATA 14.0 (StataCorp, College Station, TX, USA), and a *p*-value of less than 0.05 was considered statistically significant.

## 3. Results

In total, 133 eyes of 130 patients were included. There was a 1:1 ratio of males (n = 66) and females (n = 67), with a mean age of 59.8 ± 18.1 years (range: 19–91 years).

### 3.1. Lesion and CoNV Location

Corneal lesions were peripheral in 95/133 cases (71.4%) and central in 38 cases (28.6%). Causes of CoNV (Table 1) included microbial keratitis in 76/133 cases (57.1%), of which 43/133 (32.3%) were bacterial and 33/133 (24.8%) were herpetic. Ocular surface disorders accounted for 35/133 (26.3%) cases—these included limbal stem cell deficiency (n = 6/133, 4.5%), allergic eye disease (n = 5/133, 3.8%), previous pterygium removal (n = 6/133, 4.5%), chemical injury (n = 2/133, 1.5%), mucous membrane pemphigoid (n = 2, 1.5%), central neurotrophic ulcer (n = 2, 1.5%), exposure keratopathy (n = 1, 0.8%) and ocular surface disorders with no known cause (n = 11, 8.3%). Previous penetrating keratoplasty accounted for 11 cases (8.3%) (Table 1). In 11 cases (8.3%), a cause for CoNV was not specified.

In 86 eyes (64.7%), the CoNV was confined to one quadrant, 23 eyes (17.3%) had involvement of two quadrants, 12 (9%) had three quadrants and 12 (9%) had all four quadrants involved (Table 2). The number of quadrants involved was not different per different CoNC cause (*p* = 0.68). There was a trend for central lesions to be associated with multi-quadrant CoNV more frequently than peripheral lesions (44.7% vs. 31.6%, *p* = 0.15). For central lesions, the CoNV was fairly evenly distributed amongst four quadrants with the inferior quadrant involved in 20/38 (52.6%) cases, the superior involved in 22/38 (57.9%) cases, the nasal involved in 17/38 (44.7%) cases and temporally involved in 12/38 (31.6%) cases (Figure 2). For peripheral corneal lesions, the CoNV was co-located in the same quadrant as the lesion in most cases but with some extension into adjacent quadrants (Table 2).

### 3.2. Order of Filling of CoNV

The quadrant containing the corresponding corneal lesion was the first to fill in 84/95 (88.4%) cases, second to fill in 9/95 (9.5%) cases and third to fill in 2/95 (2.1%) cases. In cases where the first quadrant to fill did not correspond with the location of the lesion, the inferior quadrant would typically fill first (7/11 cases, 64%). For CoNV located in more than one quadrant (47 cases), a pattern of order of filling occurred: inferior, superior, nasal and then temporal (ISNT) in 61.7% of cases.

**Figure 3:**
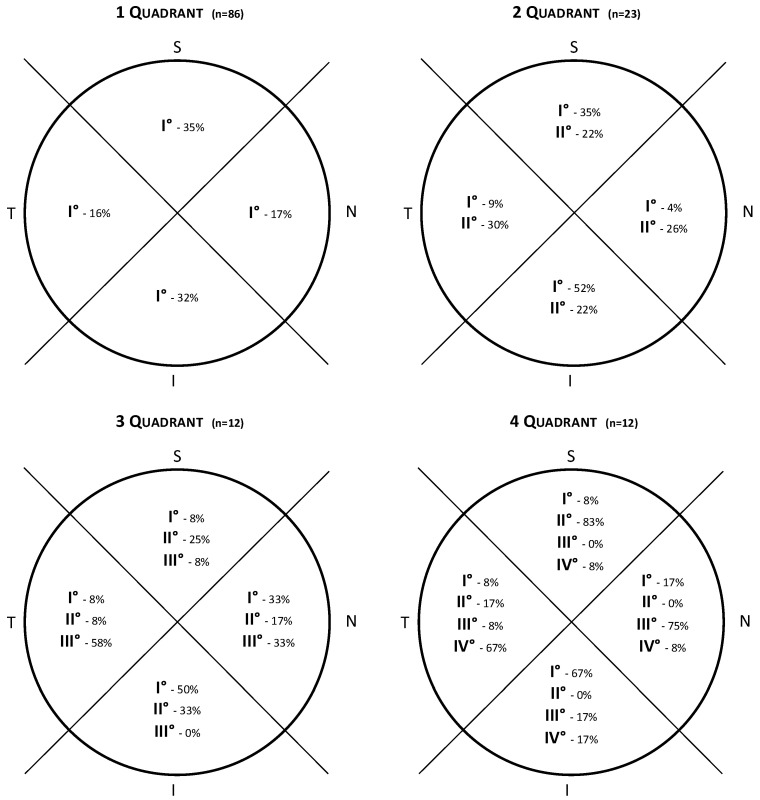
Diagram showing the filling patterns in neovascularization involving one, two, three and all four quadrants. Taking the four quadrants diagram as an example, the inferior quadrant is the first to fill in 67%, the superior quadrant fills second in 83% of cases, the nasal quadrant fills third in 75% of cases and the temporal quadrant is the last to fill in 67% of cases.

Specifically, when two quadrants of CoNV were present, the ISNT filling was respected in 69.5% of cases, 50.0% with three quadrants and 58.3% with CoNV involving four quadrants, irrespective of lesion location (Figure 3). For peripheral lesions, CoNV tended to be confined to the same quadrant; therefore, for the analysis of the filling pattern, this was limited to central lesions. Infections were associated with a more random filling pattern, respecting the ISNT rule only in 51.7% of cases (15/29), compared to the other causes, which respected the ISNT rule in 77.7% of cases (14/18). Limiting the analysis to ocular surface inflammation only, this increased to 81.8% (9/11). The inferior quadrant was significantly more likely to fill first followed by the superior and nasal followed by the temporal (Table 3).

## 4. Discussion

CoNV is associated with visual loss and is an important consideration in the management of patients, such as in corneal transplantation. Understanding angiographic filling patterns increases our understanding of angiogenesis and may improve approaches to treatment. Angiography has proven superior in detecting early signs of CoNV or in revealing more extensive CoNV than slit lamp examination only [10]. Practically, it facilitates directing treatment at likely feeder vessels such as with fine needle diathermy [6,11,12,13]. Specifically, in such cases, our protocol includes identifying the afferent vessels with angiography, cutting it with a needle at the slit lamp and cauterizing it with a thermal cautery (electrolysis needle in our practice). Our previously reported results suggest that angiographically guided FND is effective in reducing the area of CoNV [11].

Prior to assessing angiography, it is important to recognize the type of corneal lesion present and specific areas at high risk of CoNV. In peripheral corneal lesions, there is a high likelihood that CoNV is contained within the same quadrant. In contrast, with central corneal lesions, a diffuse pattern of CoNV is often present with multi-quadrant involvement often only evident with corneal angiography. Central lesions tend to be associated with larger surface areas of CoNV as compared to peripheral lesions (2.23 ± 1.5 mm^2^ vs. 2.96 ± 2.0 mm^2^). The large areas of involvement with central lesions suggest a greater, or at least less localized, angiogenic response compared to a lesion in the peripheral cornea.

Zheng et al. described MCA filling patterns in healthy corneas, in which the inferior quadrant would typically fill first, followed by the superior, nasal and then temporal quadrant (ISNT pattern of filling) [2]. The ISNT pattern, identified previously in healthy corneas, was also noted in this study—wherein approximately 60% of pathological corneas also respected the ISNT filling pattern. The reasons for such a filling pattern are not entirely clear. It is possible that vascular differences may be due to the variation in vascular supply in different quadrants. Alternatively, eyelid blinking may provide a pump-like mechanism, facilitating filling of the inferior and superior arcades to a greater extent than the lateral and medial. Anatomical variations that exist, however, might explain why this is not seen in all cases. The ISNT rule may explain some common findings, e.g., conditions, such as corneal arcus, which tends towards inferior and superior corneal margins first. It is not clear whether following or not the ISNT rule can have different prognostic implications and would require further studies to investigate.

When used in assessment of pathological corneas, this study highlights central lesions as potentially higher risk of ill-defined, larger areas of neovascularization [14]. Angiography will help delineate the location and extent of CoNV in such cases.

Although we have been able to identify key patterns in angiography of corneal pathologies, we note that several limitations arose during the analysis. Corneal angiography is a dynamic study, requiring trained technicians to obtain reproducible high-quality images, and trained clinicians to assess such images. Although observations are subjective, we aimed to minimize this by involving several independent trained observers and keeping the splitting of the quadrants standardized. Furthermore, angiographic techniques may not identify deep capillaries in the presence of masking by superficial leakage and they provide two-dimensional projections from all visualized layers rather than true three-dimensional vessel mapping. OCT-angiography (OCT-A) technologies offer a non-invasive approach that could analyze vessels throughout the entire depth of the cornea and provide simultaneous structural imaging of the cornea itself [15]. However, unlike dye-based angiography, OCT-A cannot differentiate afferent vs. efferent vessels.

We appreciate that anterior segment angiography is not readily available at many facilities; however, it is becoming an increasingly recognized tool for corneal specialists, and this study aims to allow for improved interpretation and accuracy when implementing this investigation. OCT-A technologies have the potential to provide a wider adoption, even though in the current state, those systems are less precise in capturing small vessels in CoNV complexes [16], and validation studies are needed for segmentation software [17].

The focus of this study was primarily analysis of filling patterns in pathological corneas. Anatomical variations may occur between patients; therefore, in patients with unilateral disease, using the healthy other eye as a control may be beneficial. We also note grouping of ‘central lesions’ encompasses a much wider array of pathologies as compared to the ‘peripheral lesions.’ No apparent differences were found between different causes of central lesions.

## 5. Conclusions

We identified several factors that may influence filling patterns for corneal neovascularization, including corneal lesion location, CoNV surface area and etiology of CoNV. Understanding key patterns of neovascularization may help with understanding their angiogenesis and identifying areas at higher risk, aiding in earlier detection and intervention of CoNV.

## Figures and Tables

**Table 1:** Disease and number of quadrants of corneal neovascularization (CoNV).

Cause	CoNV Quadrants Involvement (n)			
	1	2	3	4
Keratitis (n = 76)	47 (62%)	16 (21%)	8 (10%)	5 (7%)
Ocular surface disease (n = 35)	22 (63%)	6 (17%)	2 (6%)	5 (14%)
Previous graft (n = 11)	10 (91%)	-	1 (9%)	-

**Table 2:** Number of quadrants with CoNV for peripheral and central lesions.

Location	CoNV Quadrants Involvement (n)			
	1	2	3	4
Central (n = 38)	55% (21)	18% (7)	16% (6)	10% (4)
Peripheral (n = 95)	68% (65)	17% (16)	6% (6)	8% (8)

**Table 3:** Agreement of ISNT rule with respect to CoNV filling patterns for central lesions.

		Inferior	Superior	Nasal	Temporal	Total	*p*-Value
1st quadrant	Yes	15	11	7	5	38	*p* = 0.041
	No	23	27	31	33	114	
2nd quadrant	Yes	3	10	5	2	20	*p* = 0.017
	No	17	10	15	18	60	
3rd quadrant	Yes	1	1	5	2	9	*p* = 0.09 (NS)
	No	8	8	4	7	27	
4th quadrant	Yes	1	0	0	3	4	*p* = 0.046
	No	3	4	4	1	12	

NS = non-statistically significant.

## Data Availability

The data presented in this study are available on request from the corresponding author.
